# 3-Ishwarone, a Rare Ishwarane Sesquiterpene from *Peperomia scandens* Ruiz & Pavon: Structural Elucidation through a Joint Experimental and Theoretical Study

**DOI:** 10.3390/molecules181113520

**Published:** 2013-10-31

**Authors:** Fernando M. dos S. Junior, Leosvaldo S. M. Velozo, Erika M. de Carvalho, André M. Marques, Ricardo M. Borges, Ana Paula F. Trindade, Maria Isabel S. dos Santos, Ana Carolina F. de Albuquerque, Fabio L.P. Costa, Maria Auxiliadora C. Kaplan, Mauro B. de Amorim

**Affiliations:** 1 Núcleo de Pesquisas de Produtos Naturais, Universidade Federal do Rio de Janeiro, Rio de Janeiro, RJ, 21941-902, Brazil; E-Mails: fernandonppn@gmail.com (F.M.S.J.); velozo72@hotmail.com (L.S.M.V.); andrefarmaciarj@yahoo.com.br (A.M.M.); ricardo_mborges@nppn.ufrj.br (R.M.B.); nanda.20@ig.com.br (A.P.F.T.); anacarol_albuquerque@hotmail.com (A.C.F.A.); fabbioquimica@gmail.com (F.L.P.C.); makaplan@uol.com.br (M.A.C.K.); mbamorim@nppn.ufrj.br (M.B.A.); 2 Instituto de Tecnologia em Fármacos, FAR-MANGUINHOS, Fiocruz, Rio de Janeiro, RJ, 22775-903, Brazil; E-Mail: erikamc@far.fiocruz.br; 3 Departamento de Produtos Naturais e Alimentos, Faculdade de Farmácia, Universidade Federal do Rio de Janeiro, 21941-590, Rio de Janeiro, RJ, Brazil; E-Mail: sampaio@pharma.ufrj.br

**Keywords:** 3-ishwarone, structure elucidation, IR spectra simulation, NMR chemical shift calculation, molecular modeling

## Abstract

3-Ishwarone, (**1**), a sesquiterpene with a rare ishwarane skeleton, was isolated from *Peperomia scandens* Ruiz & Pavon (Piperaceae). Its structure was unambiguously determined by 1D- and 2D-NMR and infrared analyses, as well as by comparative theoretical studies which involved calculations of ^13^C-NMR chemical shifts, using the Density Functional Theory (DFT) with the mPW1PW91 hybrid functional and Pople’s 6-31G(d) basis set, and of vibrational frequencies, using the B3LYP hybrid functional and triple ζ Dunning’s correlation consistent basis set (cc-pVTZ), of (**1**) and three of its possible diastereomers, compounds **2**–**4**.

## 1. Introduction

*Peperomia* is the second largest genus of the Piperaceae family, comprising about 1,500–1,700 species, which are found in tropical regions throughout the World [1]. Although few representative species have been analyzed on the chemical point of view, *Peperomia* genus has been reported to be also the second most abundant source of bioactive compounds within the Piperaceae family [2]. Phytochemical investigations have led into the isolation of many special metabolites such as acylcyclohexane-1,3-diones, alkaloids, cinnamic acid derivatives, chromanes, chromenes, chromones, aldehydes, lignoids, flavonoids, meroterpenes, phenylpropanoids, phenols, polyketides, quinones, secolignans and sesquiterpenes [3,4,5,6,7,8]. Thus, probably for that reason, many of these plants have been used in folk medicine to treat many conditions [9,10]. *Peperomia scandens*, popularly known as German peperomia (“alemã”) or false philodendron, is an herbaceous perennial and succulent herb, which is native to the Caribbean, Mexico, and Central America [11]. *P. scandens* is well distributed in Rio de Janeiro city, easily found in public markets for sale and commonly used for ornamental purposes.

**Figure 1:**
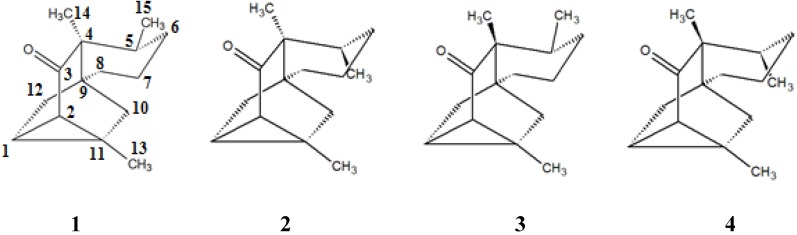
Four possible diastereomers of 3-ishwarone, (1*R*,2*S*,4*S*,5*R*,9*R*,11*R*)-3-ishwarone (configuration 1, the same stereochemistry proposed by Lago *et al.* [12]), (1*R*,2*S*,4*S*,5*S*,9*R*,11*R*)-3-ishwarone (configuration 2), (1*R*,2*S*,4*R*,5*R*,9*R*,11*R*)-3-ishwarone (configuration 3) and (1*R*,2*S*,4*R*,5*S*,9*R*,11*R*)-3-ishwarone (configuration 4), respectively.

In this work, we report the isolation from the fresh aerial parts of *P. scandens* Ruiz & Pavon of the rare but already known sequiterpene 3-ishwarone (**1**, Figure 1) [12]. Its structure was unambiguously determined by usual analysis of experimental data and from theoretical simulations of its ^13^C-NMR and IR spectra. 

## 2. Results and Discussion

Compound **1** was isolated as a white amorphous solid, whose molecular formula was defined as C_15_H_22_O based on ([M]^+^ *m/z* 218) by LREIMS. The calculated hydrogen deficiency index of 5 and the absence of olefinic carbons suggested the presence of a tetracyclic skeleton compound. Due to the excess of overlapping signals between δ 1.15–1.67 ppm and at δ 1.86–1.97 ppm in the ^1^H-NMR spectrum in CDCl_3_, both carbon and hydrogen NMR spectra were also record at C_6_D_6_ and compared to the literature data [12]. Like the known 3-ishwarone, its ^1^H- and ^13^C-NMR spectra (Table 1) showed signals related to fifteen carbon atoms, attributable to three methyls, five methylenes, three methines, three quaternary carbons and one carbonyl group. 

To reliably assign the ^1^H-NMR spectra of the compound **1**, 2D experiments such as COSY, HSQC, HMBC were carried out as the methylene group doublets at δ 1.99 ppm (*J* = 12.3 Hz) and at δ 1.66 ppm (*J* = 12.4 Hz) were assigned as H-10a and H-12b, respectively. Due to the several overlapping regions between 0.85–1.40 ppm in the ^1^H-NMR spectrum, some of the overlapped methylene hydrogen multiplets were only assigned by using 2D techniques as shown in the Table 1.

**Table 1:** ^1^H and ^13^C-NMR spectral data (δ/ppm) for 3-ishwarone in C_6_D_6_ and CDCl_3_.

Atom number	C_6_D_6_				CDCl_3_	
	δ ^1^H (multiplicity, *J/*Hz)	δ ^13^C ^a^	δ ^13^C ^b^	NOESY	δ ^1^H (multiplicity, J/Hz)	δ ^13^C
1	1.14 (1H, dd, *J* = 7.2 and 2.8 Hz)	29.7 (1)	29.9	--	1.64 (1H, dd, *J* = 3,8 and 9,0 Hz)	30.5
2	1.53 (1H, d, *J* = 7.3 Hz)	37.9(1)	37.9	--	1.51 (1H, d, *J* = 2,8 Hz)	38.2
3	--	212.5 (4)	213.4	--	--	215.0
4	--	49.3 (0)	49.9	--	--	49.3
5	1.77 (1H, dqd, *J* = 18.7, 6.6 and 3.9 Hz)	31.7 (1)	31.8	H-15	1.91 (1H. m)	31.3
6	a: 1.05–1.01 (1H, m) b: 1.24–1.17 (1H, m)	31.5 (2)	31.5	--	a: 1.38 (1H, m)	31.1
					b: 1.22 (1H, m)	
7	a: 1.24–1.21 (1H, m) b: 1.35–1.31 (1H, m)	23.5 (2)	23.6	--	a: 1.54 (1H, m)	23.1
					b: 1.40 (1H, m)	
8	a: 0.89 (1H, m) b: 1.39–1.35 (1H, m)	32.2 (2)	32.2	H-8b H-8a	a: 1.60 (1H, m)	31.9
					b: 1.19 (1H, m)	
9	--	43.0 (0)	43.1	--	--	42.9
10	a: 1.99 (1H, d, *J* = 12.3 Hz) b: 0.93 (1H, d, *J* = 12.3 Hz)	39.5 (2)	39.5	H-10a H-10b	a: 2.27 (1H, d , *J* = 12,39 Hz)	39.5
					b: 1.35 (1H, d , *J* = 12,31 Hz)	
11	--	29.8 (0)	30.0	--	--	30.8
12	a: 1.10 (1H, dd, *J* =12.4, 2.6 Hz) b: 1.66(1H, d, *J* = 12.4 Hz)	35.1 (2)	35.1	H-12b H-12a; H-14	a: 1.91 (1H, d , *J*=12,72 Hz)	34.9
					b: 1.49 (1H, d , *J* = 12,14 Hz)	
13	0.95 (3H, s)	19.4(3)	19.5	--	1.29 (3H, s)	19.5
14	0.89 (3H, s)	11.8(3)	12.0	H-12b	0.91 (3H, s)	11.5
15	1.27 (3H, d, *J* = 6.7 Hz)	17.5 (3)	17.7	H-15	1.05 (3H, d , *J* = 6,6 Hz)	17.1

^a^ Carbon type, as determined from DEPT-135 spectrum, is shown in parenthesis at the right of their respective chemical shift: quaternary (0), methine (1), methylene (2), methyl (3), carbonyl (4); ^b^ Lago *et al.* [12] (500 and 125 MHz, δ, C_6_D_6_).

The four quaternary carbon atoms and the carbonyl group positions were suggested by the 2D spectra correlations. The absence of hydrogen bearing carbon atoms in the HSQC associated to the HMBC long range correlations determined the position of the quaternary carbon atoms in the structure. The HMBC correlations between H-14 and C-3, C-4 at δ 212.5 and δ 49.3 ppm, respectively, as well as between H-1 and C-3 supported the carbonyl C-3 assignment. The NOESY space correlations between H14 and H12b suggests that relative stereochemistry for this molecule is the same proposed for 3-ishwarone; however, the NOESY sensibility for this space correlation is quite low (see Supplementary Materials) so that it seems appropriate to obtain further information to confirm the relative configuration for this molecule. In order to confirm the relative stereochemistry attributed to this product and its identity with 3-ishwarone (1), isolated for the first time from the leaves of *P. oreophila* Hensch (Piperaceae) by Lago and Co-workers [12] two additional studies were performed: comparisons of the theoretically calculated ^13^C-NMR and (fingerprint region) infrared spectra for the four diastereomers of 3-ishwarone generated by configuration inversions at carbons 4 and 5 (Figure 1) with those obtained experimentally. As far as we know the successful use of such a joint methodology has never been used in elucidating structures of such a class of sesquiterpenes [13].

It must be stressed that in doing so we aim to find out if these configuration inversions can or cannot induce easily observable changes in both ^13^C-NMR and IR spectra and thereby avoid incorrect assignment of relative configurations. As the first step of both studies, the conformationally rigid structures of these diastereomers were constructed and had their geometries optimized (with MMFF94 force-field) using the free molecular editor and visualizer program Avogadro v.1.1.0 [14]. These geometries were then re-optimized and submitted to ^13^C-NMR and IR calculations using Density Functional Theory (DFT) with the GAUSSIAN09 series of programs [15] as described in the following paragraphs.

The ^13^C chemical shift calculations were performed by a Gauge Included Atomic Orbitals-Hybrid Density Functional Theory (GIAO-HDFT) scaling factor calculation procedure previously published by some of us [16] at the GIAO/mPW1PW91/6-31G(d)//mPW1PW91/6-31G(d) [17] level of theory: namely geometry optimizations using the Perdew-Wang 1991 exchange functional as modified by Adamo and Barone combined with Perdew and Wang’s 1991 gradient-corrected correlation functional [18,19], with the 6-31G(d) Pople basis set [20], followed by absolute isotropic ^13^C magnetic shielding constants calculations for the four diastereomers (σ_i_, i = 1 to 15) and TMS (σ_0_) as internal reference using GIAO approximation at the same level of theory (GIAO/mPW1PW91/6-31G(d)). The predicted ^13^C-NMR chemical shifts, δ_i_ = σ_0_ – σ_i_, were then scaled by a factor obtained from linear correlation between the calculated and experimental chemical shifts of a pool of adequately chosen compounds [21], as δ_scal_ = a × δ_i_ + b. Comparison of the theoretical ^13^C chemical shifts obtained for the four possible diastereomers of 3-ishwarone with experimental data (Table 2) shows mean absolute (MAD) and root-mean-square (RMSD) deviations which allows discarding the diastereomers 2 and 3, these deviations came principally from carbon 5 and methyls 14 and 15, which are directly involved at configuration inversions. 

**Table 2:** Comparison of the theoretical ^13^C-NMR chemical shifts for the four possible diastereomers of 3-ishwarone with experimental data:

Atom number	Conf. 1 δ_esc_	Conf. 2 δ_esc_	Conf. 3 δ_esc_	Conf. 4 δ_esc_	EXP
**1**	30,69	29,54	31,05	30,43	**30,4**
**2**	38,10	36,57	36,74	38,24	**38,1**
**3**	212,46	211,57	211,70	212,35	**215**
**4**	49,94	50,18	50,25	50,04	**49,3**
**5**	32,76	37,42	37,60	32,70	**31,3**
**6**	31,17	27,82	27,84	31,10	**31,1**
**7**	24,09	20,27	20,18	23,94	**23,1**
**8**	33,20	33,50	33,33	33,06	**31,9**
**9**	42,45	40,64	40,78	42,48	**42,9**
**10**	39,68	41,41	42,53	41,03	**39,5**
**11**	30,63	31,23	29,81	31,09	**30,8**
**12**	35,98	37,38	36,47	34,57	**34,9**
**13**	20,51	20,66	20,61	20,51	**19,5**
**14**	13,62	23,72	23,63	13,67	**11,5**
**15**	17,38	19,45	19,50	17,35	**17,1**
**MAD**	0,84	2,89	2,90	0,86	
**RMS**	1,12	4,05	4,05	1,16	

**Figure 2:**
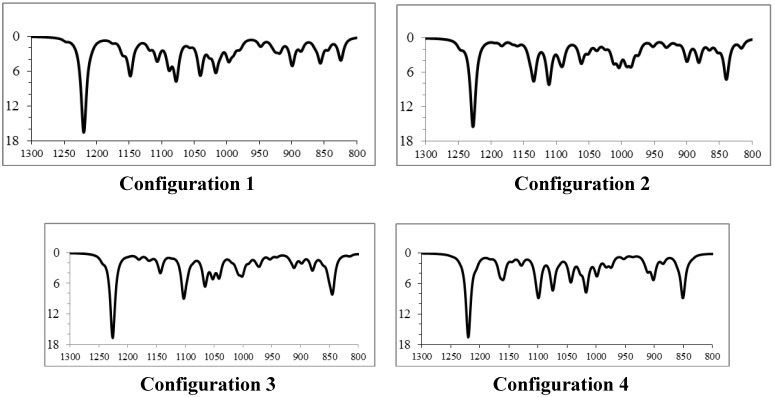
Simulated infrared spectra (fingerprint region) of the four possible diastereomers of 3-ishwarone.

**Figure 3:**
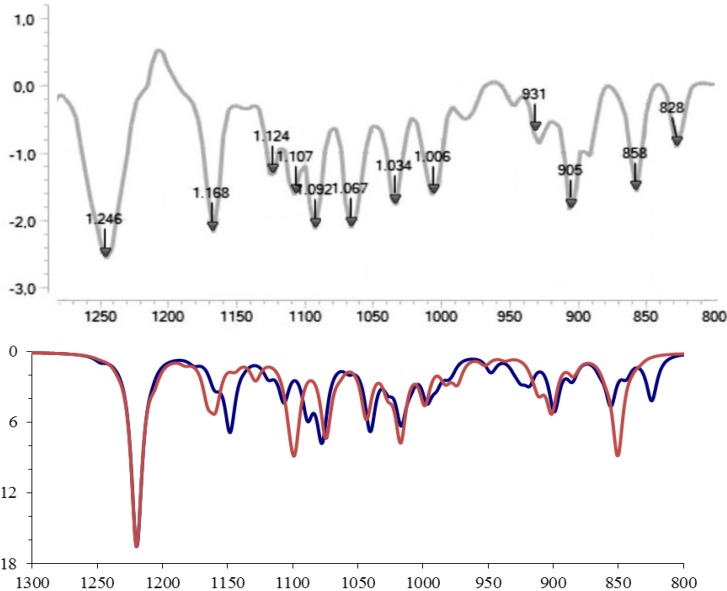
Comparison of the superposition of simulated infrared spectra (fingerprint region) of diastereomers 1 (blue) and 4 (red) (bottom) with experimental data (top).

Next we consider the comparison of the (fingerprint region) infrared spectrum obtained experimentally for the 3-ishwarone, in CHCl_3_ solution, with those simulated theoretically for the four diastereomers previously analyzed (Figure 2 and Figure 3). Vibrational frequencies were calculated through Density Functional Theory (DFT) model using the B3LYP, Beck’s three-parameter hybrid functional [22] which uses the non-local correlation provided by LYP functional [23] and VWN functional III for local correlation [24], with cc-pVTZ Dunning’s correlation consistent triple-zeta basis set [25], and corrected by an appropriated scaling factor [26]. Infrared spectra were simulated using Lorentzian band shapes and appropriate band width (5–10 cm^−1^) as follows [27]:

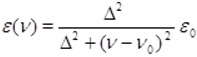
(1)


Comparison of the theoretical (fingerprint region) infrared spectra obtained for the four possible diastereomers of 3-ishwarone with experimental data (Figure 2, Figure 3, and Table 3) shows that configuration 1 has the best agreement with experimental data. Note that the main differences between the vibrational frequencies are in the region at 800-900 cm^−1^, arising from normal modes of vibration of the methyls 14 and 15 (see Supplementary Materials).

**Table 3:** Comparison of the fingerprint vibrational frequencies obtained theoretically (configuration 1) and experimentally for 3-Ishwarone:

Exp. (cm^−1^)	Theor. (cm^−1^)	*Δ_teor-exp_* (cm^−1^) ^a^
828	824	4 [0,5]
858	856	2 [0,2]
905	899	6 [0,6]
931	930	1 [0,1]
1006	997	9 [0,9]
1034	1017	17 [1,6]
1067	1040	27 [2,5]
1092	1078	14 [1,3]
1107	1089	18 [1,6]
1124	1107	17 [1,5]
1168	1148	20 [1,7]
1246	1220	26 [1,2]
	MAD=	13 [1,2]

^a^ values in brackets presents the percentage deviation in relation to calculated value.

## 3. Experimental

### 3.1. General

Sílica gel (230–400 mesh, Merck, Darmstadt, Germany) was used for column chromatographic separations while silica gel 60 PF254 (Merck) was used for analytical TLC.

### 3.2. GC-FID Analysis

Qualitative and quantitative analysis were carried out on a GC-MS-QP2010 PLUS Shimadzu apparatus (Shimadzu, Kyoto, Japan) with a DB-1MS fused silica capillary column (30 m × 0.25 mm × 0.25 µm film thickness). The operating temperatures used were: injector 260 °C, detector 290 °C and column oven 60 °C up to 290 °C (3 °C/min). Helium at 1.0 mL min^−1^ was used as a carried gas. The percentages of the compounds were obtained by GC- FID analysis.

### 3.3. GC-MS Analysis

Qualitative analysis was carried out on a GC-MS QP 5000 Shimadzu instrument with a ZB-5MS fused silica capillary column (30 m × 0.25 mm × 0.25 µm film thickness) under the experimental conditions recorded for GC-FID analysis.

### 3.4. NMR Measurements

The NMR spectra were recorded on a Brüker AVANCE-400 (400 MHz for ^1^H and 100 MHz for ^13^C) spectrometer (Brüker Spectrospin, Billerica, MA, USA) using 5 mm sample tubes, chloroform-d and benzene-d_6_ (99.9% D, CIL, Tewksbury, MA, USA) as solvents. The chemical shifts were expressed relative to TMS. The chemical shifts are expressed as δ values in parts per million (ppm) and the coupling constants (*J*) are given in hertz (Hz). The 2D experiments (^1^H-^1^H COSY, HSQC, HMBC, NOESY) were performed using standard Brüker micro programs. Gradient selections were used in all 2D techniques.

### 3.5. INFRARED Measurements

Infrared analyses were carried out on a ReactIR 15 (Mettler Toledo, Columbus, OH, USA) FTIR-ATR spectrophotometer with Attenuated Total Reflectance (ATR) technique, using a 4 mg/mL solution in chloroform.

### 3.6. Plant Material

Aerial parts of *Peperomia scandens* Ruiz & Pavon were collected in Rio de Janeiro, RJ, Brazil in 2003. Dr. Elsie Franklin Guimarães performed identification of this plant material and a voucher specimen (RB 323492) was deposited in the Herbarium of the Botanical Garden Research Institute from Rio de Janeiro, Brazil.

### 3.7. Extraction and Isolation

The fresh aerial parts of *P. scandens* (1 kg) were subjected to hydrodistillation on a Clevenger-type apparatus for 2 h, to yield 750 mg of a pale yellow crude oil, which was immediately submitted to analysis through GC-FID and GC-MS. 735 mg of this oil was submitted to column chromatography on silica gel eluted with a gradient of Hex/EtOAc yielding compound 1 (280 mg), which proved to be practically pure by GC-FID and GC-MS analyses. Melting point: 62–64 °C (literature 62–64 °C, CAS register 28895-15-0), carried out on a Fisatom 430 (Fisatom, São Paulo, Brazil). Specific Rotation [α]_D_^30^: +78° (c 0,35 CHCl_3_), carried out on a Jasco Brasil P-200 polarimeter (Jasco Brasil, São Paulo, Brazil).

## 4. Conclusions

Based on extensive analyses of experimental data and on the quite perfect agreement between calculated and experimental (fingerprint region) infrared spectra and ^13^C-NMR chemical shifts, the structure of a natural product isolated from aerial parts of *P. scandens* was unambiguously determined as 3-ishwarone, previously isolated by Lago *et al.* from another *Peperomia* species, *P. oreophila*. Its absolute configuration is under investigation in our laboratories.

## Supplementary Materials

Supplementary materials including additional IR and NMR spectral data can be accessed at: http://www.mdpi.com/1420-3049/18/11/13520/s1.
