# Supplementary material for: 3-Ishwarone, a Rare Ishwarane Sesquiterpene from Peperomia scandens Ruiz & Pavon: Structural Elucidation through a Joint Experimental and Theoretical Study
Source: Molecules. 2013 Oct 31;18(11):13520–9. doi: 10.3390/molecules181113520 (PMC6269709; doi:10.3390/molecules181113520)

Supplementary Materials

Table S1. Comparison of the finger-print vibrational frequencies obtained theoretically and experimentally for 3-Ishwarone.

	**Config 1**		***Config 2***		***ConFigure 3***		***ConFigure 4***	
**Exp. (cm^{-1})**	**Theor. (cm^{-1})**	**$\Delta_{teor\text{-}exp}$ (cm^{-1}) [a]**	**Theor. (cm^{-1})**	**$\Delta_{teor\text{-}exp}$ (cm^{-1}) [a]**	**Theor. (cm^{-1})**	**$\Delta_{teor\text{-}exp}$ (cm^{-1}) [a]**	**Theor. (cm^{-1})**	**$\Delta_{teor\text{-}exp}$ (cm^{-1}) [a]**
828	824	4 [0,5]	839	11 [1,3]	844	16 [1,9]	850	22 [2,6]
858	856	2 [0,2]	881	23 [2,6]	879	21 [2,4]	884	26 [3,0]
905	899	6 [0,6]	899	6 [0,7]	898	7 [0,8]	901	4 [0,4]
931	930	1 [0,1]	927	4 [0,4]	911	20 [2,1]	911	20 [2,1]
1006	997	9 [0,9]	985	21 [2,1]	971	35 [3,5]	998	8 [0,8]
1034	1017	17 [1,6]	1003	31 [3,0]	1001	33 [3,2]	1017	17 [1,6]
1067	1040	27 [2,5]	1061	6 [0,6]	1041	26 [2,4]	1043	24 [2,2]
1092	1078	14 [1,3]	1090	2 [0,2]	1052	40 [3,7]	1074	18 [1,6]
1107	1089	18 [1,6]	1111	4 [0,4]	1066	41 [3,7]	1098	9 [0,8]
1124	1107	17 [1,5]	1134	10 [0,9]	1103	21 [1,9]	1128	4 [0,4]
1168	1148	20 [1,7]	1183	15 [1,3]	1143	25 [2,1]	1159	9 [0,8]
1246	1220	26 [2,1]	1227	19 [1,5]	1226	20 [1,6]	1219	27 [2,2]
	**MAD=**	**13 [1,2]**	**MAD=**	**13 [1,2]**	**MAD=**	**25 [2,4]**	**MAD=**	**16 [1,5]**

[a] values in brackets presents the percentage deviation in relation at calculated value.

Figure S1. ^{13}C-NMR spectrum of 3-ishwarone in C_6D_6 solution.

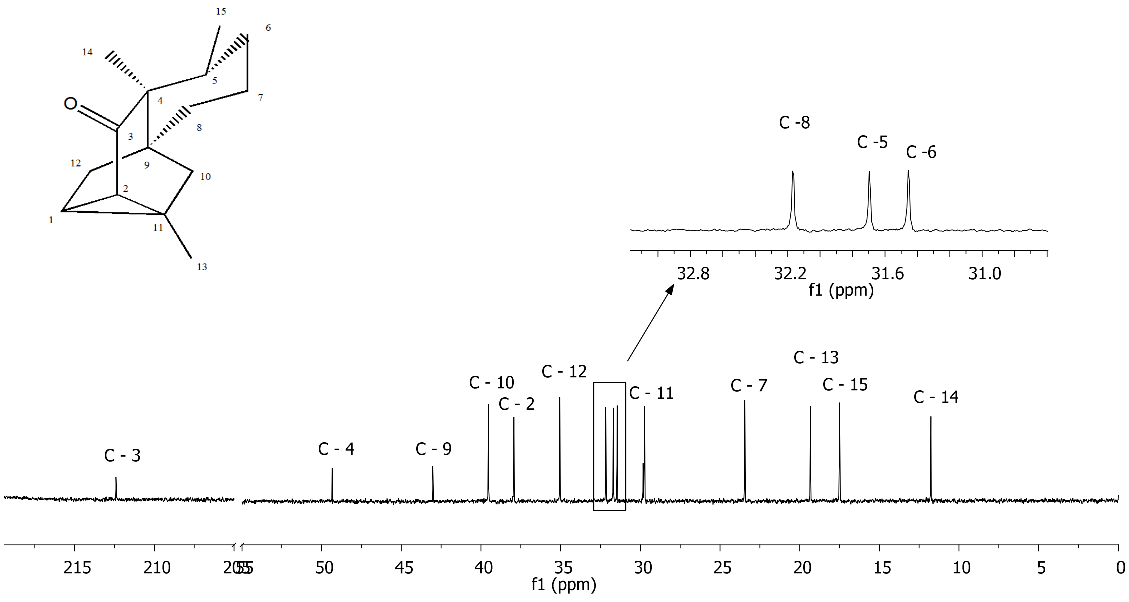

Figure S2. ^{1}H-NMR spectrum of 3-ishwarone in C_6D_6 solution.

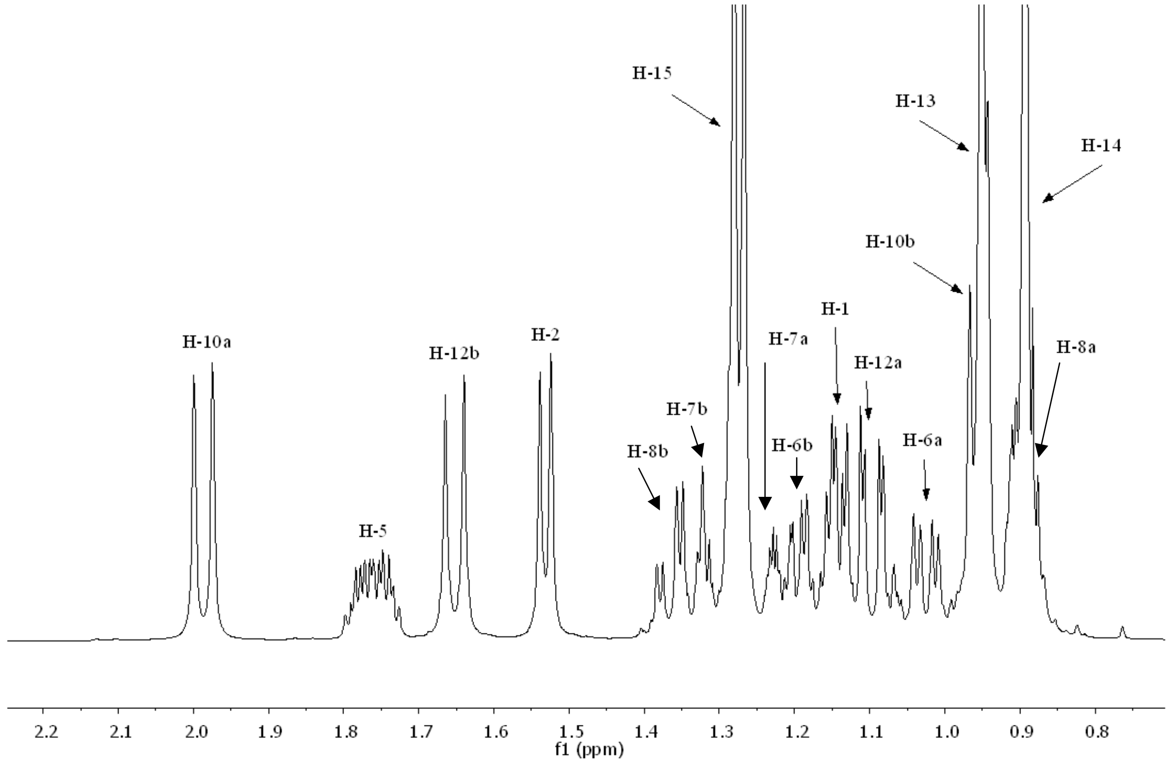

Figure S3. ^{1}H-NMR spectrum of 3-ishwarone in $CDCl_3$.

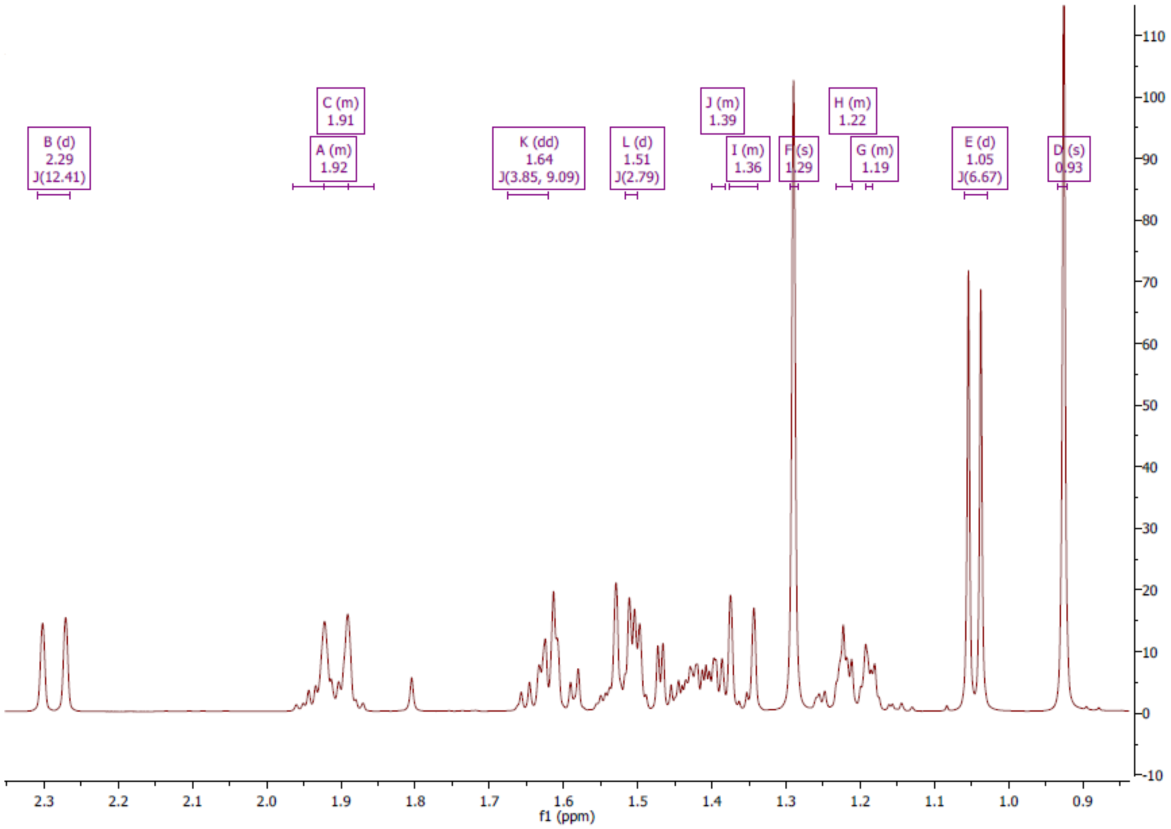

Figure S4. ^{13}C-NMR spectrum of 3-ishwarone in $CDCl_3$.

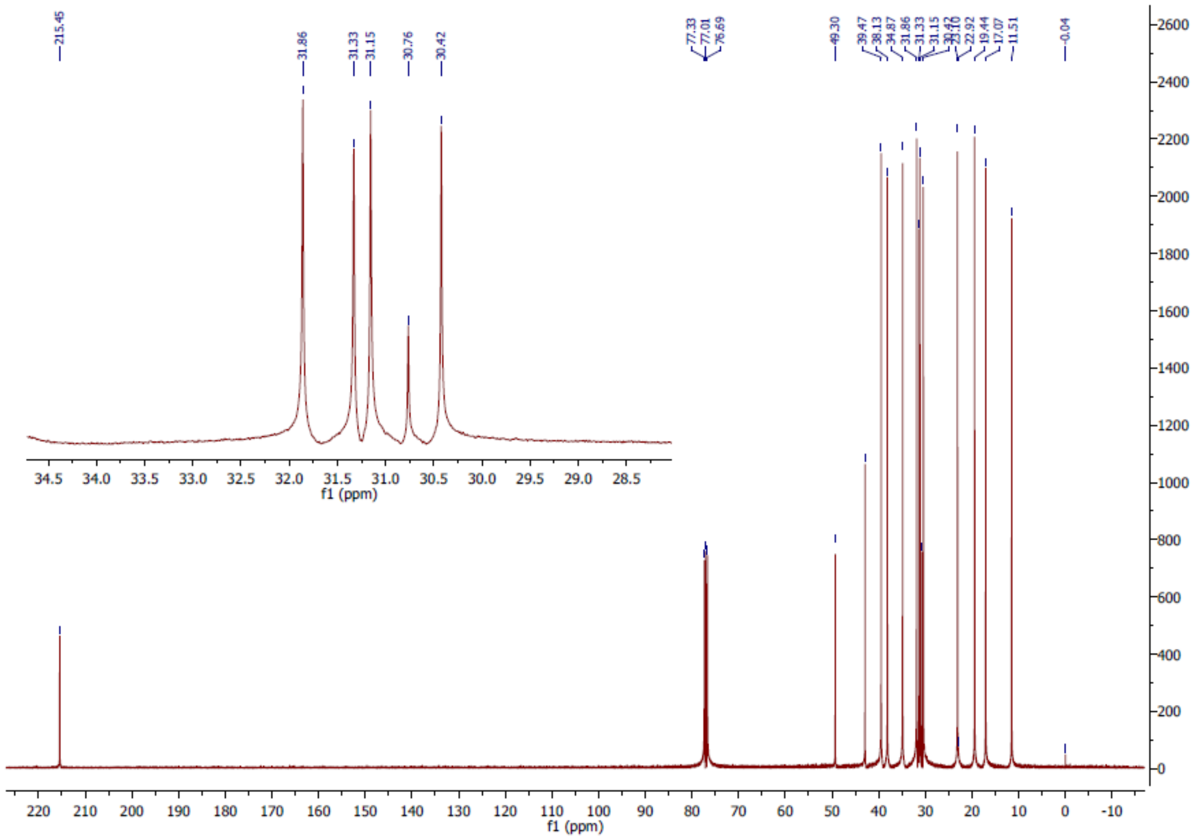

Figure S5. COSY ^{1}H-^{1}H NMR spectrum 3-ishwarone in $CDCl_3$.

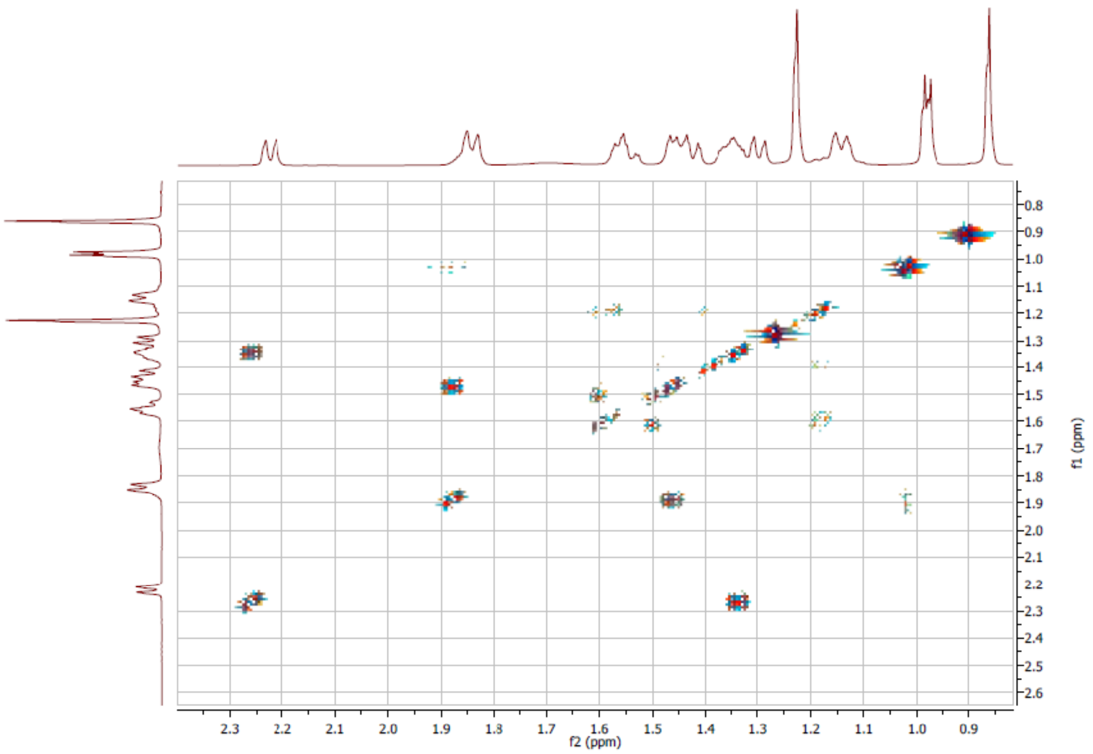

Figure S6. HSQC ^{1}H-^{13}C-NMR spectrum of 3-ishwarone in $CDCl_3$.

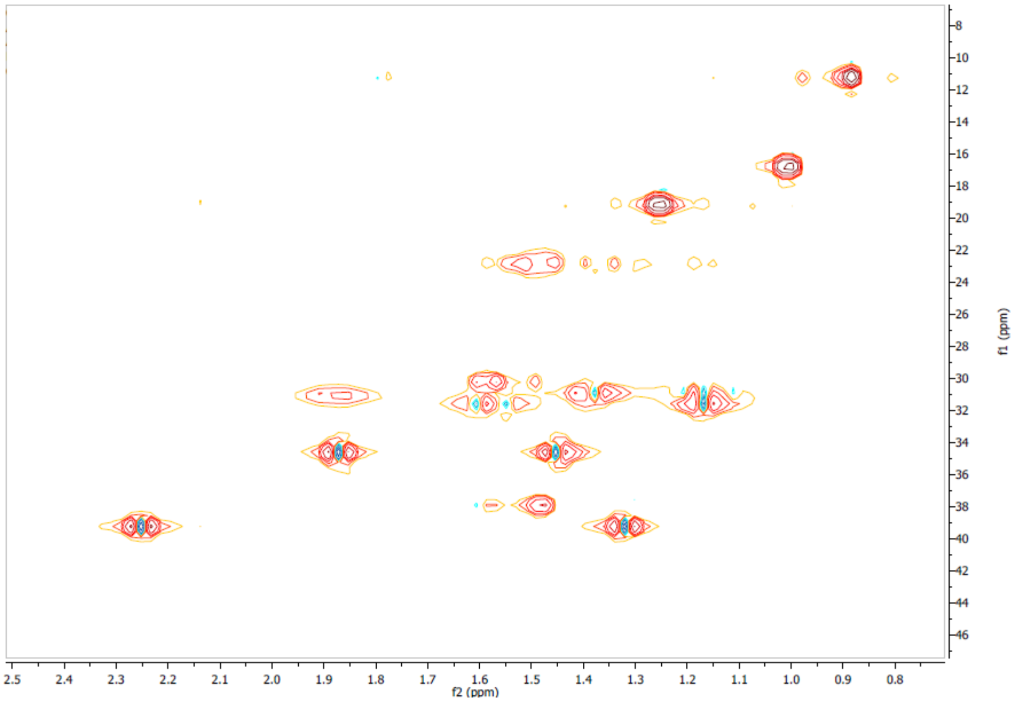

Figure S7. COSY ^{1}H-^{1}H-NMR spectrum 3-ishwarone in C_6D_6.

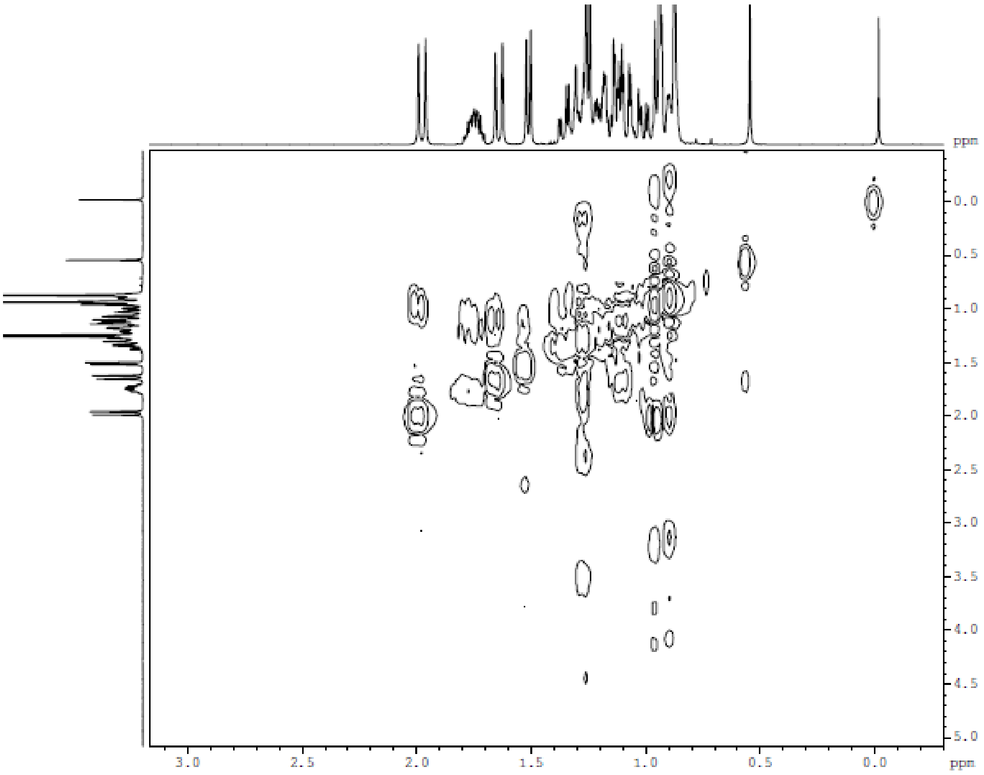

Figure S8. HSQC ^{1}H-^{13}C-NMR spectrum of 3-ishwarone in C_6D_6.

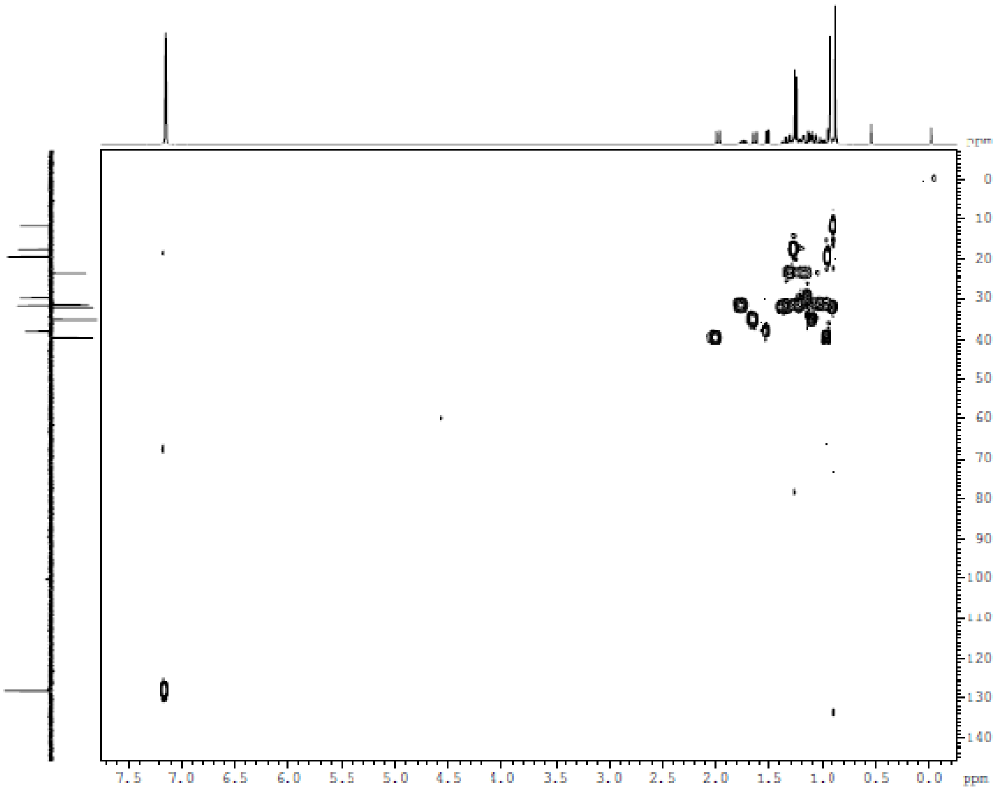

Figure S9. NOESY spectrum of 3-ishwarone in C_6D_6 (symmetrized with COSY-like, Apodization function with TRAF 1 Hz, in MNova program).

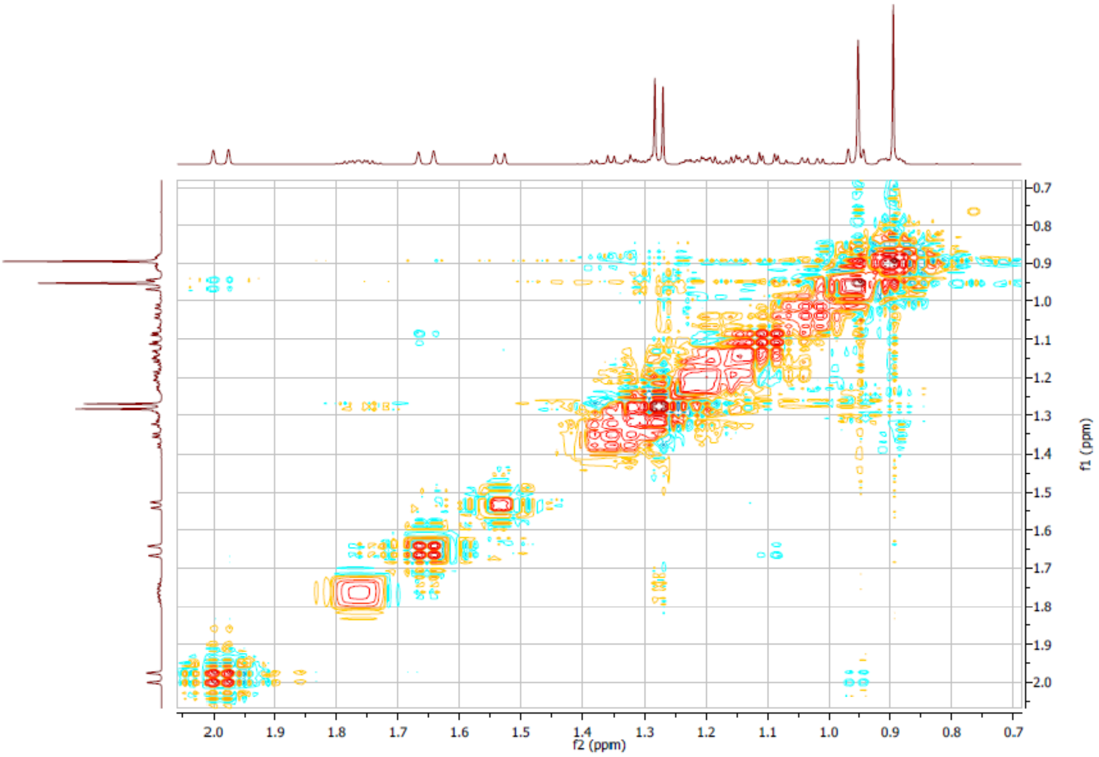

Figure S10. IR spectrum of 3-ishwarone in $CHCl_3$.

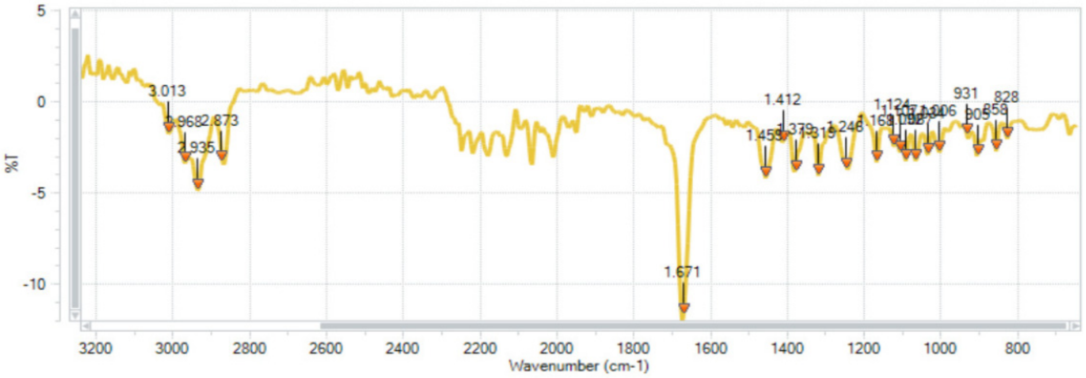

Figure S11. Chromatogram of 3-ishwarone under the experimental conditions recorded for GC-MS analysis.

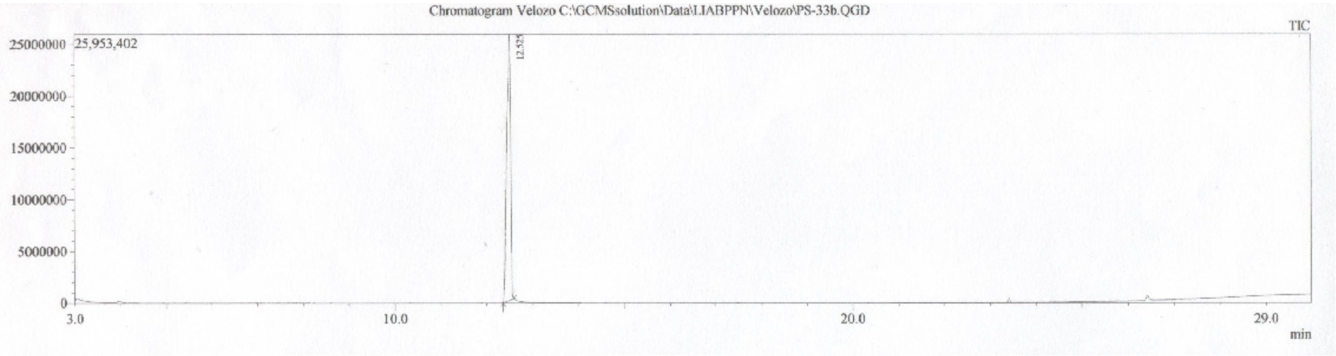

Figure S12. Mass Spectra of 3-ishwarone under the experimental conditions recorded for GC-MS analysis.

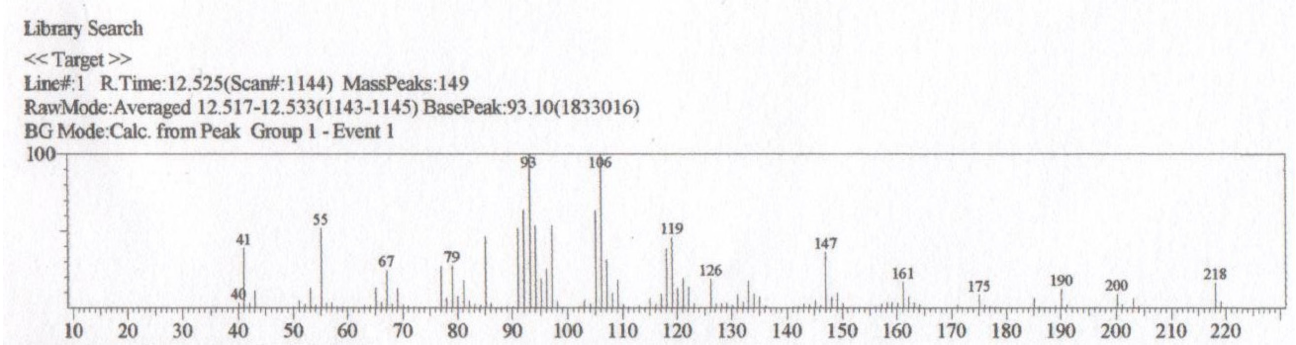

Supplement: Supplementary file 1 [file molecules-18-13520-s001.pdf]
